# Long‐Term Industrial Emissions Exposure and Cognitive Impairment in Older U.S. Adults: A Longitudinal Cohort Study

**DOI:** 10.1002/hsr2.73185

**Published:** 2026-09-03

**Authors:** Gabrielle E. Husted, Susan L. Cassels, Elizabeth S. Ackert, Barbara B. Bendlin

**Affiliations:** ^1^ Department of Geography University of California‐Santa Barbara Santa Barbara California USA; ^2^ Division of Geriatrics and Gerontology, Department of Medicine University of Wisconsin‐Madison Madison Wisconsin USA

**Keywords:** ADRD, brain health, disadvantage, geospatial, industrial emissions

## Abstract

**Background:**

The demographic shift toward an older global population is increasing the burden of Alzheimer's Disease and Related Dementias (ADRD). While age is the strongest risk factor, environmental toxicants are increasingly recognized as contributors to cognitive decline. Plastics and rubber manufacturing facilities emit neurotoxic chemicals, yet their relationship to ADRD risk remains understudied. This study assesses whether exposure to plastics/rubber industry emissions is associated with ADRD risk and whether sociodemographic factors influence this relationship.

**Methods:**

We linked geocoded Environmental Protection Agency (EPA) Toxic Release Inventory (TRI) data with Panel Study of Income Dynamics (PSID) records and Neighborhood Change Database (NCDB) Census data. Historical exposure to plastics/rubber emissions and time‐varying sociodemographic characteristics were measured from 1990 to 2017, leveraging PSID's longitudinal structure to capture residential mobility. ADRD risk was defined as a positive AD8 screen (≥ 2 endorsed cognitive challenges) in 2017, 2019, 2021, or 2023. Logistic regression models estimated ADRD risk as a function of average total pounds of chemicals released from the nearest TRI facility to each individual's census tract of residence over time, adjusting for individual and neighborhood characteristics.

**Results:**

Moderate emissions exposure was significantly associated with elevated ADRD risk (OR = 1.34), independent of income and neighborhood disadvantage. Neighborhood disadvantage was also strongly predictive of ADRD risk.

**Conclusions:**

Environmental emissions may represent a modifiable risk factor for ADRD and contribute to disparities in cognitive aging.

## Key Points

- Long‐term exposure to emissions from plastics and rubber manufacturing facilities is associated with increased risk of Alzheimer's Disease and Related Dementias (ADRD), independent of individual socioeconomic factors and neighborhood disadvantage.
- Emissions intensity, rather than proximity alone, is associated with ADRD risk, highlighting the importance of measuring environmental exposure using facility‐specific emissions data.
- Neighborhood disadvantage is a strong independent predictor of ADRD risk, underscoring the importance of structural and environmental determinants of cognitive aging.

## 1 Introduction

Phthalates—chemicals used to soften plastics and stabilize plastic products—have become pervasive since their widespread adoption in the mid‐20th century. They are present in flooring, food packaging, medical devices, and consumer products, entering the body through ingestion, inhalation, and dermal absorption [1]. Exposure is socially patterned, with low‐income and marginalized populations facing higher burdens due to differences in housing, exposure to industrial emissions, occupational environments, and product use [2, 3]. This uneven distribution is not incidental but reflects the structural forces of environmental racism: Black children face elevated lead exposure [4]. Native American communities bear disproportionate impacts from mining activity [5]. Asian, Hispanic, and Black Americans experience greater exposure to poor air and drinking water quality [6, 7]. These exposures compound to produce higher toxic body burdens in communities of color [8] and researchers increasingly attribute these findings to structural racism embedded in housing, land use, and environmental regulation rather than to chance. Among the chemical exposures of concern, phthalates warrant particular attention given their emerging neurotoxic profile. Although traditionally classified as endocrine disruptors, phthalates are increasingly recognized as neurotoxicants [9]. Human and animal studies link higher phthalate exposure to impaired memory, reduced processing speed, and altered neurological function. Proposed mechanisms—including oxidative stress, mitochondrial dysfunction, and chronic neuroinflammation—are consistent with biological pathways implicated in Alzheimer's disease and related dementias (ADRD) [10]. Despite these concerns, environmental contributions to cognitive aging remain understudied.

Alzheimer's disease accounts for 60%–80% of dementia cases and is a progressive neurodegenerative disease defined by abnormal proteinopathies (amyloid plaques and neurofibrillary tangles), with a prolonged silent phase before eventual cognitive decline, behavioral changes, and physical challenges [11, 12, 13]. The 2020 economic toll reached $305 billion [14, 15] though this doesn't capture broader societal repercussions or the loss of quality time with loved ones. Approximately 6.9 million Americans aged 65 and older have Alzheimer's dementia, with an additional 200,000 experiencing younger‐onset dementia [12]. Alzheimer's disease mortality has increased significantly, with deaths more than doubling between 2000 and 2021—a 141% rise [12]. While demographic shifts toward longer lifespans contribute to rising ADRD rates, proper understanding and access to risk‐minimizing behaviors could help plateau these trends.

ADRD pathology accumulates years before clinical diagnosis. While age and genetics are unmodifiable, twelve established modifiable risk factors—including low education, hypertension, hearing loss, obesity, smoking, and air pollution—account for about 40% of the global dementia burden [16]. Reduced environmental exposures are associated with reductions in neuropathological damage, supporting prevention efforts [17].

Environmental exposures are shaped by broader social and structural factors [18, 19]. Industrial plastics production and waste are unevenly distributed, often concentrated in historically marginalized communities [20, 21, 22, 23]. Residents living near plastics and rubber manufacturing facilities face elevated exposure to phthalates and related neurotoxic chemicals through air and environmental contamination. These patterns reflect longstanding environmental inequities and may contribute to disparities in cognitive health. However, most prior research linking environmental exposures to dementia risk has focused on general air pollution measures rather than specific industrial sources, limiting understanding of how particular sectors contribute to cognitive impairment risk.

One route through which phthalates may drive these neurotoxic effects is the gut microbiome. After absorption, phthalates can reach the gastrointestinal tract and alter microbial composition and metabolic function. Experimental evidence shows that exposure to diethylhexyl phthalate (DEHP) reduces Clostridium sensu stricto and increases Lachnoclostridium, leading to elevated p‐cresol—a microbial metabolite associated with neurodevelopmental and inflammatory disorders [24]. This dysbiosis is relevant because the gut–brain axis plays a critical role in regulating immune signaling, neurotransmitter production, and neuroinflammation. Perturbations in microbial composition can increase permeability of the gut and blood–brain barriers, triggering release of proinflammatory cytokines such as Tumor necrosis factor alpha [25]. Although gut disruption can arise from diet, antibiotics, or chronic illness, environmental chemicals are increasingly recognized as key drivers [26, 27], and the EPA's emphasis on cumulative “chemical and non‐chemical stressors” further highlights the importance of considering how social inequities compound biological vulnerability along this pathway [28].

Several additional gaps remain in our understanding of geo‐social determinants of ADRD risk. Few population‐based studies have examined long‐term exposure to industrial chemical emissions at fine geographic scales, and even fewer have accounted for residential mobility across the life course. This is important because long term exposure, rather than exposure at a single point in time, may more accurately reflect environmentally mediated risk. In addition, the environmental justice dimensions of cognitive aging remain underexplored, despite evidence that disadvantaged communities are disproportionately exposed to industrial hazards and experience higher burdens of chronic disease [29, 30].

This study addresses these gaps by linking longitudinal, geocoded data on industrial emissions from plastics and rubber facilities with individual cognitive outcomes over nearly three decades. Using data from the Panel Study of Income Dynamics, the Environmental Protection Agency Toxic Release Inventory, and U.S. Census‐derived neighborhood measures, we examine whether long‐term exposure to plastics and rubber industry emissions is associated with ADRD risk. We further assess whether sociodemographic and neighborhood factors shape this relationship; they are conceptualized here as effect modifiers of the exposure‐ADRD relationship, reflecting how structural inequities can compound or buffer the biological impact of environmental exposures on cognitive aging. ADRD risk was assessed using the AD8 Dementia Screening Interview, a brief, validated informant‐report tool shown to reliably detect early cognitive impairment when compared against clinical assessments like the Clinical Dementia Rating (CDR) and has been favorably compared to other screening tools such as the Mini‐Mental State Examination (MMSE) and Montreal Cognitive Assessment (MoCA) [31, 32]. By identifying environmental contributors to cognitive impairment, this study aims to advance understanding of modifiable risk factors [16] and inform prevention strategies to promote equitable cognitive aging.

## 2 Methods

### 2.1 Data and Sample

We used individual‐level data from the Panel Study of Income Dynamics (PSID), linked to environmental exposure data from the Environmental Protection Agency Toxic Release Inventory (TRI) and neighborhood sociodemographic data from the Neighborhood Change Database (NCDB). The PSID is a nationally representative longitudinal study that has followed U.S. families since 1968 and includes detailed socioeconomic and health information. Restricted geocoded identifiers enabled linkage of individuals to census tracts across survey waves.

Our analytic sample included PSID respondents aged 65 years or older by 2023 with valid responses to the Eight‐item Informant Interview to Differentiate Aging and Dementia (AD8), administered in 2017, 2019, 2021, and 2023. The AD8 Dementia Screening Interview is a brief, validated tool designed to detect early signs of dementia in community‐based populations [31, 32]. In the PSID, the AD8 screener consists of eight questions about cognitive and functional changes, such as difficulty with appointments, finances, or learning new tasks, making it particularly useful for identifying pre‐clinical cognitive decline (see Appendix Table 1 for the full list).

**Table 1:** Distribution of ADRD risk by year of AD8 Survey and potential observation values. Data: Panel Study of Income Dynamics (PSID), years 2017–2023, unweighted.

ADRD	2017, *n* (%)	2019, *n* (%)	2021, *n* (%)	2023, *n* (%)	2017–2023, *n* (%)
At Risk	475 (12.39%)	523 (13.64%)	501 (13.06%)	494 (12.83%)	672 (25.58%)
No Risk	1767 (46.08%)	1943 (50.68%)	2142 (55.82%)	2273 (59.05%)	1955 (74.42%)
0 [NA]	1593 (41.54%)	1368 (35.69%)	1194 (31.12%)	1082 (28.11%)	—
Total	3835	3834	3837	3849	2627

*Note:* *Row ‘0 [NA]' is referring to the individuals that did not have data on ADRD risk and were therefore not included in analysis.

ADRD risk was defined as endorsement of two or more cognitive difficulties on the AD8 in any wave. Individuals who were not administered the AD8 due to sample design or nonresponse were excluded. The final analytic sample included 2,627 respondents. Table 1 depicts the distribution of participants aged 65 and older that are considered to have ADRD risk, that are not considered to have ADRD risk, and that identify as inapplicable to the AD8 survey for each of our outcome measure years, 2017–2023.

The exposure assessment window spanned 1990–2017, allowing evaluation of long‐term exposure prior to ADRD assessment. Census tract boundaries were harmonized to 2020 definitions to ensure geographic consistency across datasets. Our analytic sample spans 6,595 unique census tracts (approximately 8% of all U.S. census tracts), reflecting the geographic distribution of PSID respondents meeting our sample criteria (age 65 + , AD8 survey respondent); see Table 5 for state‐level coverage in areas with known concentrations of plastics/rubber facilities.

### 2.2 Environmental Exposure Measures

Industrial emissions data were obtained from the EPA TRI, which tracks annual facility‐level releases of toxic chemicals to air, land, and water (See Supplemental Information Section B for an extended discussion on Toxics Release Inventory Reporting). We identified plastics and rubber manufacturing facilities and calculated annual emissions volumes and geographic proximity to each census tract.

For each year, we assigned each census tract the total pounds of chemicals released by the nearest plastics/rubber facility and calculated distance (km) between tract centroids and facilities using geographic information system methods. These annual values were linked to individuals based on residential tract and averaged across all observed years (1990–2017) to create person‐level measures of long‐term exposure (See Supplemental Information Section A). Exposure variables were categorized into percentile‐based groups to account for right‐skewed distributions (Tables 2 and 3).

### 2.3 Neighborhood Characteristics

Neighborhood socioeconomic context was measured using the NCDB, which provides harmonized census tract data across decades. We constructed a neighborhood disadvantage index using census‐based indicators reflecting socioeconomic and housing conditions (See Supplemental Information Section C for more index construct details; Appendix Figure 1 for the PCA scree plot; Appendix Figure 2 for the distribution of the resulting index). Individual exposure to neighborhood disadvantage was defined as the mean index value across all residential locations observed during the study period.

### 2.4 Individual Covariates

Covariates were selected based on prior research and the social determinants of health framework [15]. Individual‐level variables included age, sex, race, educational attainment, household income, marital status, home ownership, and residential mobility (Tables 2 and 3). Income was calculated as the average inflation‐adjusted household income percentile across observed years. Education and demographic characteristics were measured using the most recent available data. Race was operationalized using categories available in the PSID: White, Black, and all other racial groups (combined due to small subgroup sample sizes). Consistent with recommendations by well‐regarded frameworks for discussing race, we conceptualize race in this analysis as a proxy for differential exposure to systemic and structural racism rather than as a marker of inherent biological difference [33].

### 2.5 Data Linkage and Processing

PSID individual records were linked to TRI facility data and NCDB tract‐level characteristics by census tract and year. This linkage produced a longitudinal dataset capturing residential location, environmental exposures, and neighborhood characteristics over time. The analytic dataset was collapsed to the person level by summarizing exposure and covariate measures across observation years.

### 2.6 Statistical Analysis

We used descriptive statistics, bivariate tests, and logistic regression models [34] to estimate the association between plastics and rubber industry emissions and ADRD risk. Exposure was measured using both emissions volume and proximity to the nearest facility. Models were sequentially adjusted for individual sociodemographic characteristics and neighborhood disadvantage.

This study uses a longitudinal design, linking PSID respondents' industrial exposure and covariate data from 1990 to 2017 to ADRD risk assessed via the AD8 instrument, administered in 2017, 2019, 2021, and 2023. ADRD risk was defined as an indicator for whether a participant ever met AD8 criteria (≥ 2 memory challenges) in any of these four survey waves. While exposure and covariate measures capture change over time where appropriate (e.g., residence, income) and stability where appropriate (e.g., education), models were not structured as time‐varying, consistent with our focus on cumulative exposure history rather than year‐to‐year risk fluctuation.

This analysis considers two related but distinct measures of industrial exposure. ‘Emissions exposure' refers to the average annual total chemical releases (in pounds) reported by the nearest plastics/rubber TRI facility to a participant's residential census tract centroid across the observation period (1990–2017), and serves as our primary exposure measure in Models 1–4. ‘Proximity to the nearest facility' refers instead to the average distance (in kilometers) from the residential census tract centroid to the nearest plastics/rubber TRI facility over the same period, independent of the volume of emissions reported. Because the nearest facility in a given year is not necessarily the same facility with the highest emissions, these two measures can diverge: a participant may live close to a low‐emitting facility, or farther from a high‐emitting one. Model 5 incorporates both measures jointly to evaluate whether proximity contributes additional explanatory value beyond emissions volume alone.

Model 1 estimated the unadjusted association between emissions exposure and ADRD risk. Model 2 adjusted for individual sociodemographic factors. Model 3 additionally adjusted for neighborhood disadvantage. Model 4 evaluated interaction between emissions exposure and income to assess effect modification. Model 5 incorporated both emissions exposure and proximity to the nearest facility. Odds ratios for all models are presented in Table 4. Alternative model specifications using distance‐based exposure, median/tail categorization, and log‐transformed exposure are described in Supplemental Information Section D and are reported in Appendix Table [Link], [Link], [Link], [Link], [Link], [Link], [Link], [Link].

**Table 2:** Unweighted descriptive statistics (*n* = 2,627). Data: Panel Study of Income Dynamics (PSID) 1990–2023, Environmental Protection Agency Toxic Release Inventory (EPA TRI) 1990–2017, and Neighborhood Change Database (NCDB) 1990–2020.

Variable	*N*	Percentage/Mean
ADRD risk indicator (2017–2023)		
No Risk	1955	74.4%
At Risk	672	25.6%
Average Distance to Nearest Plastics/Rubber TRI Facility, km (1990–2017) *continuous*	2,627	25.2 km
Average Distance to Nearest Plastics/Rubber TRI Facility, km (1990–2017) *categorical*		
1 Near, 0.53–30.54 km (min up to 75th percentile)	1970	75.0%
2 Moderate, 30.54–50.59 km (75th‐90th percentile)	393	15.0%
3 Far, 50.59+ km (90th+ percentile)	264	10.0%
Average Exposure to Nearest Plastics/Rubber TRI Emissions, lbs (1990–2017) *continuous*	2627	86,052 lbs
Average Exposure to Nearest Plastics/Rubber TRI Emissions, lbs (1990–2017) *categorical*		
0 None, 0 lbs	1545	58.8%
1 Low, > 0–7627.52 lbs (up to 75th percentile)	425	16.2%
2 Moderate, 7627.52 lbs–75797.66 lbs (75th–90th percentile)	394	15.0%
3 High, 75797.66+ lbs (90th+ percentile)	263	10.0%
Age (2023)		
65–70 y.o.	1222	46.5%
71–80 y.o.	1088	41.4%
81–90 y.o.	259	9.9%
91+ y.o.	58	2.2%
Sex (2023)		
Male	1193	45.4%
Female	1434	54.6%
Race (2023)		
White	1754	66.8%
Black	728	27.7%
All other	145	5.5%
Wtr ever married (2023)		
Never married	141	5.4%
Current/previously married	2486	94.6%
Total moves (1990‐2017)		
*0*	695	26.5%
*1–2*	936	35.6%
*3–5*	637	24.3%
*6–9*	296	11.3%
*10* +	63	2.4%
Home ownership (1990–2017)		
Never a homeowner	168	6.4%
Current or previous homeowner	2459	93.6%
Max education level (1990–2017)		
Less than 12 years	240	9.1%
12 years or more, less than 16 years	1477	56.2%
16 years or more	910	35%
Household average income percentile (1990–2017)	2,627	54.085
Household income binary (recode of Household Average Income Percentile, 1990–2017)		
Bottom 50%	1314	50.1%
Top 50%	1313	50.0%
Mean disadvantage index, standardized (1990–2017)	2,627	−0.06
**Total sample**	2,627	

Descriptive statistics and bivariate analyses were conducted to characterize the sample and assess unadjusted associations. Statistical significance was defined as *p* < 0.10, consistent with environmental epidemiology conventions prioritizing detection and magnitude of potential risk relationships. Analyses were conducted using Stata MP 17.0, with spatial processing performed in R 4.6.0.

## 3 Results

### 3.1 Sample Characteristics

The analytic sample included 2,627 individuals aged 65 and older, of whom 25.58% screened positive for ADRD risk at any point between 2017 and 2023 (Table 2). Exposure to plastics and rubber industry emissions varied substantially across participants. The majority (58.81%) experienced no emissions exposure from the nearest TRI facility during the observation period, while 16.18% were classified as low exposure, 15.00% moderate exposure, and 10.01% high exposure. The mean distance from the participants' residential census tract centroid to the nearest plastics/rubber TRI facility was 25.20 km (median = 16.31 km), with 74.99% of participants residing within 30.54 km of a facility. Distances were calculated using the centroid of the census tract in which each participant resided at each observation period (1990–2017), updated accordingly if a participant relocated.

The sample was relatively socioeconomically advantaged. Most participants had completed at least 12 years of education (91.22%), and 93.60% had been homeowners at some point during the observation period. Average household income was slightly above the population midpoint (mean percentile = 54.09). Participants lived in neighborhoods with slightly lower‐than‐average disadvantage relative to the PSID reference population (mean standardized disadvantage index = −0.06), indicating generally favorable neighborhood socioeconomic conditions.

### 3.2 Bivariate Associations

ADRD risk differed significantly across emissions exposure categories (Table 3). Individuals in the moderate and high emissions exposure groups had the highest prevalence of ADRD risk (29.95% and 29.28%, respectively), compared to 23.56% among those with no exposure (χ^2^ *p* = 0.025). In contrast, distance to the nearest plastics/rubber TRI facility was not significantly associated with ADRD risk in unadjusted analyses (*p* = 0.753).

**Table 3:** Descriptive statistics stratified by ADRD Risk (*n* = 2,627). Unweighted chi‐squared values are reported. Data: Panel Study of Income Dynamics (PSID) 1990–2023, Environmental Protection Agency Toxic Release Inventory (EPA TRI) 1990–2017, and Neighborhood Change Database (NCDB) 1990–2020. Significance levels: *p* < 0.05 *, *p* < 0.01 **, *p* < 0.001 ***.

Variable	No ADRD Risk (%), mean (SE)	ADRD Risk (%), mean (SE)	(un‐weighted) χ^2^
ADRD risk indicator (2017–2023)			—
No Risk	100%	—	
At Risk	—	100%	
Average Distance to Nearest Plastics/Rubber TRI Facility, km (1990‐2017) *continuous*	25.1 km	25.6 km	0.669
Average Distance to Nearest Plastics/Rubber TRI Facility, km (1990–2017) *categorical*			0.753
1 Near, 0.53–30.54 km (min up to 75th percentile)	74.5%	25.5%	
2 Moderate, 30.54–50.59 km (75th–90th percentile)	75.3%	24.7%	
3 Far, 50.59+ km (90th+ percentile)	72.7%	27.3%	
Average Exposure to Nearest Plastics/Rubber TRI Emissions, lbs (1990–2017) *continuous*	95,536 (SE: 23,384)	58,461 (SE: 9933)	0.358
Average Exposure to Nearest Plastics/Rubber TRI Emissions, lbs (1990–2017) *categorical*			0.025**
0 None, 0 lbs	76.4%	23.6%	
1 Low, > 0–7627.52 lbs (up to 75th percentile)	73.4%	26.6%	
2 Moderate, 7627.52 lbs–75797.66 lbs (75th–90th percentile)	70.1%	30.0%	
3 High, 75797.66+ lbs (90th+ percentile)	70.7%	29.3%	
Age (2023)			< 0.001***
65–70 y.o.	82.5%	17.5%	
71–80 y.o.	72.7%	27.8%	
81–90 y.o.	55.6%	44.4%	
91+ y.o.	29.3%	70.7%	
Sex (2023)			0.642
Male	74.8%	25.2%	
Female	74.1%	26.0%	
Race (2023)			0.005**
White	76.3%	23.7	
Black	70.9%	29.1%	
All other	69.0%	31.0%	
Wtr ever married (2023)			0.018*
Never married	66.0%	34.0%	
Current/previously married	75.0%	25.1%	
Total moves (1990–2017)			0.005**
0	75.8%	24.2%	
1–2	75.5%	24.5%	
3–5	74.7%	25.3%	
6–9	71.0%	29.1%	
10+	55.6%	44.4%	
Home ownership (1990–2017)			0.002**
Never a homeowner	64.3%	35.7%	
Current or previous homeowner	75.1	24.9%	
Max education level (1990–2017)			< 0.001***
Less than 12 years	60.8%	39.2%	
12 years or more, less than 16 years	73.6%	26.4%	
16 years or more	79.3%	20.7%	
Household average income percentile (1990–2017)	55.0 (SE: 0.523)	43.5 (SE: 0.874)	
Household income binary (recode of Household Average Income Percentile, 1990–2017)			< 0.001***
Bottom 50%	66.4%	33.6%	
Top 50%	82.4%	17.6%	
Mean disadvantage index, standardized (1990–2017)	−0.116 (SE: 0.019)	0.089 (SE: 0.033)	
Total sample %	71.6%	28.4%	

Several sociodemographic factors were strongly associated with ADRD risk. Risk increased markedly with age, rising from 17.51% among individuals aged 65%–70% to 70.69% among those aged 91 and older (*p* < 0.001). Lower socioeconomic status was also associated with elevated ADRD risk. Individuals in the bottom half of the income distribution had substantially higher ADRD risk than those in the top half (33.56% vs. 17.59%, *p* < 0.001). Similarly, participants with fewer than 12 years of education had higher ADRD risk (39.17%) compared to those with 16 or more years of education (20.66%, *p* < 0.001).

Neighborhood context was also associated with ADRD risk. Individuals at risk of ADRD lived in more disadvantaged neighborhoods on average (mean index = 0.089) compared to those without ADRD risk (mean index = −0.116). Residential instability was associated with increased ADRD risk, with individuals experiencing 10 or more moves showing substantially higher prevalence (44.44%) compared to non‐movers (24.17%, *p* = 0.005). Race, marital history, and homeownership were associated with ADRD risk in unadjusted analyses as well.

### 3.3 Multivariable Regression Results

Multivariable logistic regression models estimating the association between plastics and rubber industry emissions and ADRD risk are presented in Table 4. In unadjusted models (Model 1), moderate and high emissions exposure were associated with significantly elevated odds of ADRD risk compared to no exposure (OR = 1.387, *p* < 0.01; OR = 1.343, *p* < 0.05, respectively). After adjustment for individual sociodemographic characteristics (Model 2), the association for moderate exposure remained statistically significant (OR = 1.341, *p* < 0.05), while the association for high exposure was attenuated and no longer statistically significant.

**Table 4:** Odds ratios from logistic regression models predicting ADRD risk. Reference categories and model fit statistics are shown. Significance levels: *p* < 0.05 *, *p* < 0.01 **, *p* < 0.001 ***.

Variable	Model 1	Model 2	Model 3	Model 4	Model 5
Average Exposure to Nearest Plastics/Rubber TRI Emissions, lbs (1990‐2017) *categorical*					
*Ref* = *0 None, 0 lbs*					
1 Low, > 0–7627 lbs (up to 75th percentile)	1.175	1.14	1.135	1.11	1.141
2 Moderate, 7627 lbs–75,797 lbs (75th–90th percentile)	1.387**	1.341*	1.339*	1.263	1.343*
3 High, 75,797+ lbs (90th+ percentile)	1.343*	1.2	1.203	1.134	1.202
Sex (2023)					
*Ref = Male*					
Female		0.9	0.904	0.927	0.904
Race (2023)					
*Ref = White*					
Black		1.062	0.936	0.997	0.924
All other		1.434	1.34	1.333	1.33
Age (2023)					
*Ref* = *65–70 y.o*.					
71–80 y.o.		2.033***	2.066***	2.079***	2.073***
81–90 y.o.		3.881***	3.986***	4.231***	3.984***
91+ y.o.		11.638***	12.418***	13.302***	12.385***
Household Average Income Percentile (1990–2017)		0.982***	0.983***		0.983***
Total Moves (1990–2017)					
*Ref* = *0 moves*					
1–2		1.188	1.193	1.19	1.194
3–5		1.186	1.175	1.202	1.172
6–9		1.378	1.355	1.417	1.354
10+		2.027*	2.026*	2.337**	2.024*
Wtr ever married (2023)					
*Ref = Never married*					
Current/previously married		0.885	0.874	0.751	0.874
Home ownership (1990–2017)					
*Ref = Never a homeowner*					
Current or previous homeowner		1.096	1.158	1.013	1.168
Max education level (1990–2017)					
*Ref = Less than 12 years*					
12 years or more, less than 16 years		0.819	0.85	0.762	0.843
16 years or more		0.845	0.875	0.697*	0.869
Mean Disadvantage Index, Standardized (1990–2017)			1.171*	1.207*	1.168*
Household Income Binary (recode of Household Average Income Percentile, 1990–2017)					
*Ref = Bottom 50%*					
Top 50%				0.550***	
Binary Income*Exposure to Emissions					
Ref = Bottom 50% income*None					
Top 50% income*Low				1.076	
Top 50% income*Moderate				1.203	
Top 50% income*High				1.183	
Average Distance to Nearest Plastics/Rubber TRI Facility, km (1990‐2017) *categorical*					
*Ref* = 1 *Near, 0.53–30.54 km (min up to 75th percentile)*					
2 Moderate, 30.54–50.59 km (75th‐90th percentile)					0.88
3 Far, 50.59+ km (90th+ percentile)					0.96
_cons	0.308***	0.485*	0.437**	0.345***	0.452*
	m1	m2	m3	m4	m5
*n*	2627	2627	2627	2627	2627
aic	2986.299	2755.262	2751.857	2772.724	2754.968
bic	3009.793	2866.86	2869.329	2907.817	2884.187
ll	−1489.149	−1358.631	−1355.929	−1363.362	−1355.484

Further adjustment for neighborhood disadvantage (Model 3) did not substantially alter the association between moderate emissions exposure and ADRD risk (OR = 1.339, *p* < 0.05), indicating that moderate emissions exposure was independently associated with increased ADRD risk. Individuals exposed to moderate emissions levels had approximately 34% higher odds of ADRD risk compared to unexposed individuals.

Models including interaction terms between emissions exposure and income (Model 4) did not reveal significant effect modification, suggesting that the association between emissions exposure and ADRD risk did not differ significantly by income level.

To distinguish emissions effects from spatial proximity, Model 5 included both emissions exposure and distance to the nearest facility. Moderate emissions exposure remained significantly associated with ADRD risk (OR = 1.343, *p*< 0.05), while distance to the nearest facility was not significantly associated with ADRD risk. This paper focuses on the results from the models measuring exposure by emissions volume, however, the results models measuring exposure by emissions proximity can be found in Appendix Table 2.

### 3.4 Other Predictors of ADRD Risk

Several individual and neighborhood characteristics were significantly associated with ADRD risk. Age was the strongest predictor, with individuals aged 91 and older having more than 13 times the odds of ADRD risk compared to those aged 65–70 (OR = 13.302, *p* < 0.001). Higher household income was associated with lower ADRD risk (OR = 0.983 per percentile increase, *p* < 0.001), and higher educational attainment was associated with lower odds of ADRD risk. Individuals with 16 or more years of education had significantly lower odds of ADRD risk compared to those with fewer than 12 years (OR = 0.697, *p* < 0.05).

Neighborhood disadvantage was independently associated with increased ADRD risk (OR = 1.171, *p* < 0.05), indicating that long‐term exposure to socioeconomically disadvantaged environments may contribute to cognitive vulnerability. High levels of residential mobility were also associated with elevated ADRD risk, with individuals experiencing 10 or more moves having approximately twice the odds of ADRD risk compared to non‐movers (OR = 2.027, *p* < 0.05).

Sex, race, marital status, and homeownership were not significantly associated with ADRD risk after adjustment for socioeconomic and neighborhood factors.

## 4 Discussion

This study examined whether long‐term exposure to plastics and rubber industry emissions is associated with ADRD risk and whether socioeconomic context modifies this relationship. We found that moderate levels of emissions exposure were significantly associated with increased ADRD risk, independent of individual socioeconomic characteristics and neighborhood disadvantage. In contrast, proximity alone was not associated with ADRD risk, and income did not significantly modify the relationship between emissions exposure and cognitive outcomes. These findings partially support our hypothesis and suggest that emissions intensity, rather than spatial proximity alone, may be the more relevant exposure metric.

The association between moderate emissions exposure and ADRD risk remained statistically significant after adjustment for age, income, education, residential mobility, and neighborhood disadvantage. Individuals with moderate exposure had approximately 34% higher odds of ADRD risk compared to those with no exposure. Although high exposure was associated with increased ADRD risk in unadjusted models, this relationship was attenuated after adjustment for socioeconomic and neighborhood factors. This attenuation suggests that social and structural determinants may confound or mediate relationships between environmental exposures and cognitive health. This is consistent with an ecological framing of environmental health, in which measured exposures operate within the microsystem — the immediate settings individuals interact with — while structural determinants, such as racialized housing policy and disinvestment, function at the macrosystem level shaping which environments individuals are exposed to in the first place [35, 36]. Under this framing, adjusting for social and structural determinants may attenuate the exposure‐outcome relationship not because the exposure is unimportant, but because it partially captures the same upstream structural forces the covariates are measuring.

Our findings align with a growing body of work linking place‐based social and environmental vulnerability to ADRD‐relevant outcomes. Prior work has found that higher social vulnerability and environmental justice burden were associated with greater cerebral blood flow variability and reduced cortical thickness among Black participants specifically, suggesting that place‐based measures may partially capture the neurobiological imprint of structural racism [37]. Similarly, researchers have found that environmental and social vulnerability, as measured by the Environmental Justice Index (EJI), were significantly elevated among racial/ethnic minority participants in a mild cognitive impairment clinical trial, though neither the EJI nor Area Deprivation Index (ADI) independently predicted cognitive decline in that sample [38]. When paired with our findings, this literature suggests that place‐based exposure measures are strongly patterned by race and structural context, but that the pathway from exposure to measurable cognitive or biomarker change may be attenuated, mediated, or masked depending on sample composition, outcome measure, and the specific index used.

The absence of a monotonic dose–response relationship warrants careful interpretation. Several mechanisms may contribute to this pattern. First, residential selection processes may influence observed associations. Individuals with greater socioeconomic resources may be more likely to relocate away from high‐emission facilities or mitigate exposure through protective resources such as healthcare access, nutrition, and housing quality. Conversely, moderate‐exposure areas may represent environments where emissions levels are sufficient to confer risk while residents face greater structural vulnerability. Second, exposure misclassification is likely inherent in proximity‐based measures. Our person‐level averaging approach does not capture peak exposures, atmospheric dispersion, or contributions from multiple facilities, which may obscure true exposure gradients. These methodological constraints are common in environmental epidemiology and may attenuate observed dose–response relationships.

Importantly, distance to the nearest plastics and rubber facility was not significantly associated with ADRD risk, suggesting that proximity alone is an insufficient proxy for exposure. Facilities vary substantially in emission volume, chemical composition, and environmental dispersion patterns. Two individuals living at similar distances from facilities may experience very different exposure levels depending on facility emissions and environmental conditions. These findings highlight the importance of incorporating emissions intensity and facility‐specific characteristics into exposure assessment rather than relying solely on spatial proximity.

Our finding that neighborhood disadvantage was a strong predictor of ADRD risk is consistent with recent large‐scale evidence. In a national cohort of over 1.6 million older veterans, there was a dose‐response relationship between Area Deprivation Index quintile and dementia risk, with veterans in the most disadvantaged neighborhoods facing a 22% higher hazard of dementia diagnosis relative to those in the least disadvantaged quintile [39]. Similarly, another study found that neighborhood disadvantage was associated with worse performance across memory, executive function, and processing speed domains in cognitively unimpaired adults, and that this relationship was partially explained by altered cortical morphological organization in frontal and temporal regions [40]. This literature supports neighborhood disadvantage as a robust and mechanistically plausible predictor of cognitive decline, reinforcing the relevance of our finding within the broader ADRD literature.

Neighborhood disadvantage emerged as a strong and consistent predictor of ADRD risk, independent of individual socioeconomic characteristics. Individuals living in more disadvantaged neighborhoods had significantly higher odds of ADRD risk, reinforcing evidence that cognitive aging is shaped by cumulative exposure to structural and environmental conditions. Disadvantaged neighborhoods may expose residents to multiple risk factors, including environmental hazards, chronic stress, limited healthcare access, and fewer health‐promoting resources. Future work could extend this measure of neighborhood disadvantage by incorporating additional structural indicators, such as historical redlining classifications or residential segregation indices, to more directly characterize the pathways linking place‐based structural inequity to cognitive health [33]. These findings support a growing body of literature emphasizing the importance of social and environmental determinants in cognitive health and dementia risk [28, 35, 41, 42, 43].

Household income was independently associated with ADRD risk but did not modify the relationship between emissions exposure and ADRD. This suggests that environmental exposures and socioeconomic disadvantage may contribute to ADRD risk through additive rather than interactive pathways. The absence of significant effect modification may also reflect limitations in income measurement or statistical power, as detecting interaction effects typically requires larger sample sizes, particularly given only 263 individuals in our highest exposure category. Future research should examine more granular measures of socioeconomic resources and wealth to better understand how structural inequality shapes vulnerability to environmental neurotoxicants.

Age was the strongest predictor of ADRD risk, consistent with established literature identifying aging as the primary risk factor for dementia. Other socioeconomic indicators, including race, education, and homeownership, were associated with ADRD risk in unadjusted analyses but were attenuated in multivariable models, likely reflecting shared variance with income and neighborhood disadvantage. These findings underscore the importance of considering multiple dimensions of socioeconomic context when examining cognitive health outcomes.

Several limitations should be considered when interpreting these findings. First, exposure assessment relied on proximity to TRI facilities and facility‐reported emissions data, which may not accurately reflect individual‐level exposure. This approach does not account for pollutant dispersion, indoor exposure environments, or individual behaviors influencing exposure. Second, the PSID analytic sample represented approximately 8% of U.S. census tracts and showed limited overlap with areas containing TRI facilities (see Table 5), which may reduce generalizability and statistical power. Underrepresentation of highly industrialized regions may attenuate observed associations. Third, ADRD risk was measured using the AD8 screening instrument, which identifies cognitive impairment risk but does not provide clinical diagnoses (see Appendix Table 1 for the list of questions utilized in the PSID AD8). Fourth, the observational design limits causal inference, and unmeasured confounding may influence observed associations.

**Table 5:** Approximate PSID observational coverage in U.S. states with known high concentrations of plastics/rubber facilities.

Known high concentrations of plastics industries (Appalachia and Gulf States)	Available observations (approximation, due to restricted geocoded data)
Louisiana	Low
Texas	High
Alabama	Low
Mississippi	Low
Florida	High
Indiana	Mid
Illinois	Mid
Ohio	High

TRI reporting requirements introduce additional measurement considerations [44]. Facilities report emissions only when exceeding regulatory thresholds, and some facilities reporting zero emissions may still handle substantial chemical quantities through recycling or treatment. These reporting structures may result in incomplete capture of environmental exposures, particularly in lower‐emission areas. Future studies should differentiate facility types and incorporate more refined exposure assessment methods, including atmospheric dispersion modeling and biomarker‐based exposure measures.

Despite these limitations, this study contributes novel evidence linking emissions from plastics and rubber manufacturing to ADRD risk using longitudinal, geocoded data spanning nearly three decades. By incorporating residential mobility and neighborhood context, this analysis provides a life‐course perspective on environmental contributions to cognitive health. These findings extend prior research linking air pollution to dementia risk by examining source‐specific industrial emissions rather than aggregate pollution measures.

These results have important implications for environmental health and dementia prevention. Industrial emissions represent modifiable environmental risk factors that may contribute to cognitive decline at the population level. Regulatory policies aimed at reducing emissions from plastics and rubber manufacturing facilities may have downstream benefits for cognitive health, particularly in communities disproportionately burdened by industrial activity. Addressing neighborhood disadvantage and environmental inequities may also represent important pathways for reducing dementia risk.

Future research should build on these findings not only through more precise exposure metrics, including biomarker‐based measures and dispersion modeling, but also through methodologically distinct approaches to this question. Qualitative and community‐engaged research in high‐exposure areas could elucidate pathways, such as chronic stress, healthcare access, or occupational co‐exposure, that are difficult to capture through administrative data alone. Quasi‐experimental designs leveraging facility openings, closures, or regulatory changes could also help strengthen inferences, beyond what observational proximity‐based designs allow. Studies integrating environmental, biological, and social data will be critical for understanding mechanisms linking environmental toxicants to neurodegeneration. Expanding geographic representation and improving exposure assessment will help identify populations at greatest risk and inform targeted prevention strategies.

In conclusion, long‐term exposure to plastics and rubber industry emissions was associated with increased ADRD risk at moderate exposure levels, independent of socioeconomic and neighborhood factors. Neighborhood disadvantage was also a strong predictor of ADRD risk, highlighting the importance of structural and environmental determinants of cognitive aging. These findings underscore the need to consider environmental exposures within broader socio‐environmental frameworks and support efforts to reduce industrial emissions and address structural inequities to promote healthy cognitive aging.

## Author Contributions

**Gabrielle E. Husted:** conceptualization, methodology, formal analysis, data curation, visualization, writing – review and editing, writing – original draft, investigation, validation. **Susan L. Cassels:** conceptualization, methodology, supervision, writing – review and editing. **Elizabeth S. Ackert:** conceptualization, methodology, supervision, writing – review and editing, resources. **Barbara B. Bendlin:** conceptualization, supervision, writing – review and editing, methodology.

## Funding

The authors have nothing to report.

## Ethics Statement

This study used secondary data from the Panel Study of Income Dynamics (PSID), accessed under a restricted data use agreement. All analyses, models, figures, and tables derived from the restricted PSID data were reviewed and approved by the PSID data administration in accordance with the terms of the data use agreement prior to manuscript preparation. These data were merged with publicly available data from the U.S. Environmental Protection Agency's Toxics Release Inventory (TRI) and the U.S. Census Bureau. No new data were collected, and the study involved secondary analysis of existing data. All data were handled in accordance with applicable data use agreements and institutional requirements for the protection of participant confidentiality.

## Conflicts of Interest

The authors declare no conflicts of interest.

## Transparency Statement

The lead/corresponding author, Gabrielle Husted, affirms that this manuscript is an honest, accurate, and transparent account of the study being reported; that no important aspects of the study have been omitted; and that any discrepancies from the study as planned (and, if relevant, registered) have been explained.

## Supporting information


Supporting File 1

Supporting File 2

Supporting File 3

Supporting File 4

Supporting File 5

Supporting File 6

Supporting File 7

Supporting File 8

Supporting File 9

## Data Availability

Some of the data used in this analysis are derived from Restricted Data Files of the PSID, obtained under special contractual arrangements designed to protect the anonymity of respondents. These data are not available from the authors. Persons interested in obtaining PSID Restricted Data Files should contact PSIDHelp@umich. edu.
