# Supplementary material for: Long‐Term Industrial Emissions Exposure and Cognitive Impairment in Older U.S. Adults: A Longitudinal Cohort Study
Source: Health Sci Rep. 2026 Sep 3;9(9):e73185. doi: 10.1002/hsr2.73185 (PMC13542189; doi:10.1002/hsr2.73185)
Supplement: Supplementary file 1 [file HSR2-9-e73185-s001.docx]

|  | **Model 1** | **Model 2** | **Model 3** | **Model 4** |
| --- | --- | --- | --- | --- |
| **Variable** |  |  |  |  |
| **Average Exposure to Nearest Plastics/Rubber TRI Emissions, lbs (1990-2017) *categorical*** |  |  |  |  |
| *Ref = Low (≤ 50th)* |  |  |  |  |
| Mid (50-90th) | 1.274* | 1.236* | 1.232 | 1.193 |
| High (>90th) | 1.347* | 1.197 | 1.201 | 1.116 |
|  |  |  |  |  |
| **Sex (2023)** |  |  |  |  |
| *Ref = Male* |  |  |  |  |
| Female |  | 0.897 | 0.9 | 0.923 |
|  |  |  |  |  |
| **Race (2023)** |  |  |  |  |
| *Ref = White* |  |  |  |  |
| Black |  | 1.062 | 0.936 | 0.994 |
| All other |  | 1.431 | 1.338 | 1.329 |
|  |  |  |  |  |
| **Age (2023)** |  |  |  |  |
| *Ref = 65-70 y.o.* |  |  |  |  |
| 71-80 y.o. |  | 2.031*** | 2.064*** | 2.079*** |
| 81-90 y.o. |  | 3.879*** | 3.983*** | 4.240*** |
| 91+ y.o. |  | 11.529*** | 12.296*** | 13.197*** |
|  |  |  |  |  |
| **Household Average Income Percentile (1990-2017)** |  | 0.982*** | 0.983*** |  |
|  |  |  |  |  |
| **Total Moves (1990-2017)** |  |  |  |  |
| *Ref = 0 moves* |  |  |  |  |
| 1-2 |  | 1.187 | 1.192 | 1.188 |
| 3-5 |  | 1.188 | 1.176 | 1.204 |
| 6-9 |  | 1.367 | 1.344 | 1.408* |
| 10+ |  | 2.018* | 2.017* | 2.334** |
|  |  |  |  |  |
| **Wtr Ever Married (2023)** |  |  |  |  |
| *Ref = Never married* |  |  |  |  |
| Current/previously married |  | 0.887 | 0.876 | 0.752 |
|  |  |  |  |  |
| **Home Ownership (1990-2017)** |  |  |  |  |
| *Ref = Never a homeowner* |  |  |  |  |
| Current or previous homeowner |  | 1.095 | 1.156 | 1.012 |
|  |  |  |  |  |
| **Max Education Level (1990-2017)** |  |  |  |  |
| *Ref = Less than 12 years* |  |  |  |  |
| 12 years or more, less than 16 years |  | 0.82 | 0.851 | 0.762 |
| 16 years or more |  | 0.844 | 0.875 | 0.696* |
|  |  |  |  |  |
| **Mean Disadvantage Index, Standardized (1990-2017)** |  |  | 1.170* | 1.208** |
|  |  |  |  |  |
| **Household Income Binary (recode of Household Average Income Percentile, 1990-2017)** |  |  |  |  |
| *Ref = Bottom 50%* |  |  |  |  |
| Top 50% |  |  |  | 0.549*** |
|  |  |  |  |  |
| **Binary Income*Exposure to Emissions** |  |  |  |  |
| *Ref = Bottom 50% income*None* |  |  |  |  |
| Top 50% income*Mid |  |  |  | 1.11 |
| Top 50% income*High |  |  |  | 1.227 |
|  |  |  |  |  |
| _cons | 0.308*** | 0.488* | 0.440** | 0.346*** |
| n | 2627 | 2627 | 2627 | 2627 |
| aic | 2985.423 | 2754.234 | 2750.87 | 2769.841 |
| bic | 3003.043 | 2859.959 | 2862.468 | 2893.186 |
| ll | -1489.711 | -1359.117 | -1356.435 | -1363.92 |
| Appendix Table 3: Pounds (lbs) exposure categorized by median and tail distribution. | | | | |
