# Supplementary material for: Long‐Term Industrial Emissions Exposure and Cognitive Impairment in Older U.S. Adults: A Longitudinal Cohort Study
Source: Health Sci Rep. 2026 Sep 3;9(9):e73185. doi: 10.1002/hsr2.73185 (PMC13542189; doi:10.1002/hsr2.73185)
Supplement: Supplementary file 2 [file HSR2-9-e73185-s002.docx]

# A. Exposure Variable Construction

Using 2020 TIGER/Line census tract shapefiles, we obtained tract centroids computed as the geometric center of each tract polygon using the largest polygon component to ensure a single representative point per tract. All spatial data were projected to the Albers Equal Area Conic coordinate system (EPSG:5070 – NAD83/Conus Albers) to ensure accurate distance calculations across the continental United States.

We excluded (n = 3) individuals who lived in non-continental US locations (e.g., Alaska, Hawaii, US territories, and foreign county identifiers) during any observation year to ensure comparable geographic exposure assessment, as the density and distribution of manufacturing facilities differs substantially in these regions.

Using nearest neighbor analysis with the *sf* and *nngeo* packages in R, we identified the closest facility in any emission category to every census tract centroid and calculated the Euclidean distance in kilometers.

Although census tract centroids are spatially fixed, the set of nearby TRI facilities changes over time as facilities open, close, or vary in their annual reporting activity. Consequently, the nearest facility to a given tract centroid—and the corresponding distance and emission values—may differ across years. To capture this temporal variation, we calculated year-specific distances between each census tract centroid and the nearest active plastics/rubber TRI facility for each year from 1990 through 2017, using Euclidean distance (in kilometers). This approach allows exposure measures to reflect dynamic changes in the industrial landscape rather than assuming static proximity. Each person-year observation was assigned the distance and total chemical releases from the nearest facility to their census tract centroid in that year. We then averaged these annual values across all observation years to create person-level continuous variables representing mean distance (in km) and mean exposure (in pounds) to the nearest plastics/rubber TRI facility over the study period (1990-2017).

Our analytic sample spans 6,595 unique census tracts (approximately 8% of all U.S. census tracts), reflecting the geographic distribution of PSID respondents meeting our sample criteria (age 65+, AD8 survey respondent).

The following example demonstrates our data processing approach for a single individual using simulated data. Due to the restricted nature of our geocoded PSID data, we present this illustration with fabricated values while maintaining methodological accuracy:

*Longitudinal data for one person*

| Tract ID | Year | Nearest TRI ID | Distance (km) | Total Releases (lbs) |
| --- | --- | --- | --- | --- |
| 101 | 1990 | TRI123 | 4.5 | 12,000 |
| 101 | 1991 | TRI123 | 4.5 | 11,500 |
| 101 | 1995 | TRI789 | 2.1 | 34,000 |
| 101 | 2000 | TRI789 | 2.1 | 32,000 |
| 202 | 2003 | TRI456 | 6.2 | 5,000 |

*Summary data for one person*

| Tract IDs | Mean Distance (km) | Mean Total Releases (lbs) |
| --- | --- | --- |
| 101, 202 | 3.88 | 22,375 \ 18,900 |

Given the right-skewed distribution of both our average annual values of emission exposure (pounds released by nearest plastics/rubber TRI and distance to nearest plastics/rubber TRI), with a concentration of observations at lower values (median distance = 16.31 km), median emissions = 0 lbs) and a long tail extending to higher values (maximum distance = 299.70 km, maximum emissions = 30,800,000 lbs), we categorized both measures of exposure into groups-based percentiles.

Person level average pounds of exposure from nearest plastics/rubber TRI across all years in which they are observed (1990-2017):

- 0 None, 0 lbs
- 1 Low, > 0-7,627 lbs (greater than zero up to 75th percentile)
- 2 Moderate, 7,627 lbs - 75,797 lbs (75th-90th percentile)
- 3 High, 75,797+ lbs (90th+ percentile)

Person level average distance to plastics/rubber TRI (regardless of emitter level) across available years (1990-2017) in kilometers. (minimum value is 0.53 km):

1. Near, 0.53-30.55 km (min up to 75th percentile)
2. Moderate, 30.55-50.59 km (75th-90th percentile)
3. Far, 50.59+ km (90th+ percentile)

This approach ensured adequate sample sizes for each exposure category while maintaining meaningful exposure contrasts. Models using alternative categorization, in addition to log transformation, are reported in Appendix Tables 3-6; results remain largely unchanged.

# B. Extended Discussion on Toxics Release Inventory Reporting (TRI)

Facilities must report to the TRI if they exceed chemical quantity thresholds and meet facility criteria. Standard reporting thresholds are 25,000 pounds for manufacturing or processing and 10,000 pounds for otherwise using TRI-listed chemicals. However, Persistent, Bioaccumulative, and Toxic (PBT) chemicals, such as lead, certain plastics, and some PFAS, have much lower thresholds (e.g., 100 pounds). Beyond chemical thresholds, facilities must also have 10+ full-time equivalent employees and operate under a covered North American Industry Classification System (NAICS) industry code. Federal facilities are exempt from NAICS requirements. Facilities exceeding any applicable threshold must report. Additionally, facilities meeting reporting criteria must submit TRI reports even when reporting *zero* emissions, as the inventory tracks not only releases but also pollution prevention activities, recycling, energy recovery, and waste treatment practices. Thus, a facility may appear in TRI data with zero reported emissions while still managing substantial quantities of TRI-listed chemicals through recycling or other waste management activities that prevent environmental release. These reporting thresholds and the inclusion of facilities with zero emissions may create systematic differences in exposure measurement across geographic areas, potentially contributing to the observed pattern where very low exposure areas may have incomplete capture of industrial activity while some facilities in our reference category (zero emissions) may actually be managing significant chemical quantities through pollution prevention measures. Future research should further differentiate types of industrial activity within the TRI, such as comparing proximity to plastics/rubber recycling facilities versus production facilities, to better understand how varying modes of chemical management relate to ADRD risk.

# C. Explanation of Neighborhood Disadvantage Index

To measure neighborhood disadvantage, we constructed a composite census tract disadvantage index using eight census tract-level variables from the NCDB from years 1990-2017. We followed the framework and logic of the Neighborhood Atlas Area Deprivation Index, however, our selection of these 8 variables was determined by data availability, coverage, and alignment with our other variables we use in the PSID^1^. We chose to create our own Census Tract Disadvantage Index in order to have a comparison among our sample of participants, remaining hyper-local in our methodology.

Several variables required transformation prior to analysis. We calculated the proportion of elderly residents as the ratio of residents aged 65 and older to the total tract population, excluding tracts with zero or missing total population. We derived the proportion of non-white residents by subtracting the percentage of white residents from 100%. To create a low education indicator consistent with our disadvantage framework, we subtracted the percentage of college-educated residents from 100, so that higher values indicated lower educational attainment. The final set of variables included: poverty rate, unemployment rate, proportion elderly, proportion non-white, proportion of men never married, proportion of women never married, proportion with low college education, and proportion of renter-occupied housing units.

After geocoding PSID participants to census tracts and merging with NCDB data (1990-2017), we employed principal component analysis (PCA) to create a single disadvantage index using only the tracts where PSID participants in our analytic sample resided. This approach ensured the index optimally weighted the contribution of each variable based on the variation present within neighborhoods actually experienced by our study population. Prior to analysis, we assessed the appropriateness of PCA using the Kaiser-Meyer-Olkin (KMO) measure of sampling adequacy^2^. The overall KMO value was 0.7150, meeting the recommended threshold of 0.70 and indicating good sampling adequacy. Individual variable KMO values ranged from 0.6119 (proportion of men never married) to 0.8314 (proportion of renter-occupied housing), all surpassing the minimum acceptable level of 0.50.

The PCA was based on 5,640 unique census tracts with data pooled across multiple years (1990-2017), resulting in 52,803 complete tract-year observations. We examined the correlation matrix and scree plot to ensure variables were appropriately intercorrelated and supported a single-factor solution. As shown in Appendix Figure 1, the scree plot indicates a strong one-factor structure underlying the census tract disadvantage index, with a steep drop between the first and second eigenvalues. This supports the use of the first principal component as a summary measure of neighborhood disadvantage. The resulting index provides a time-varying, tract-specific measure of disadvantage, with each tract receiving a score for each year of available data.

The first principal component explained 41.63% of the total variance across all eight variables, reflecting a coherent underlying construct of neighborhood disadvantage. Component loadings showed that the poverty rate (0.4090) and proportion of women never married (0.4014) contributed most strongly to the index, followed by proportion non-white (0.3946), proportion of men never married (0.3656), renter-occupied housing (0.3461), and unemployment rate (0.3611). The low college education variable showed a moderate loading (0.2725). Notably, the proportion of elderly residents had a negative loading (-0.2404), suggesting that neighborhoods with higher elderly populations tended to have lower disadvantage scores. This pattern is consistent with our sample geography, which included substantial representation from Florida and California, where elderly populations often reside in more affluent, established communities.

We generated standardized disadvantage index scores using the first principal component, with higher scores indicating greater neighborhood disadvantage. The standardized index (mean = 0, standard deviation = 1) provides an interpretable measure where positive values indicate above-average disadvantage and negative values indicate below-average disadvantage relative to all census tracts in the sample. This continuous variable is well-suited for multilevel modeling and allows for meaningful comparisons across census tracts. Appendix Figure 2 presents the density distribution of the standardized neighborhood disadvantage index. The distribution illustrates variation in neighborhood disadvantage across 5,640 unique census tracts (52,803 complete observations over years 1990-2017) represented in the PSID sample, with a roughly normal shape and some skewness toward higher disadvantage. The index captures a continuum of disadvantage consistent with the socio-demographic composition of neighborhoods.

The PCA results demonstrated strong construct validity. The single-factor solution, high proportion of variance explained, adequate sampling measures, and theoretically consistent factor loadings all support the use of this composite index as a valid measure of neighborhood disadvantage in our study context.

# D. Additional Model Specifications

The main models we reported on use emissions exposure measured in pounds by the nearest plastics/rubber TRI, and we categorize them based on meaningful percentiles (min value up to 75th percentile, 75th-90th percentile, 90th+ percentile). Model 5 also takes into account the distance to the nearest emitter (of any poundage amount), and these are categorized similarly.

Throughout this process, we explored various approaches to capture changes in residential location over time and link them to plastics/rubber industry data. We develop five sets of alternative models based on different exposure measures:

1. **Distance-based replication**: The same five models presented in this paper, but using kilometers to nearest plastics/rubber TRI facility (any emission level) rather than pounds emitted (Appendix Table 2)
2. **Pounds median/tail**: Pounds exposure categorized by median and tail distribution (Appendix Table 3)
3. **Distance median/tail**: Kilometers exposure categorized by median and tail distribution (Appendix Table 4)
4. **Log-transformed pounds**: Log-transformed pounds exposure divided into quartiles (Appendix Table 5
5. **Log-transformed distance**: Log-transformed kilometers exposure divided into quartiles (Appendix Table 6)

Model output tables for all five alternative model sets are provided in the Appendices as indicated above.

Key findings:

**Model Set 1 (Distance-based replication, Appendix Table 2):** No exposure measures are significant. Age, total moves, average income percentile, and disadvantage index remain significant. Sex, race, marital status, homeownership, education, and the income×exposure interaction are not significant.

**Model Set 2 (Pounds median/tail, Appendix Table 3):** Similar pattern to our main model—mid-level exposure to emissions shows elevated OR for ADRD risk, with greater attenuation after adding covariates. Age, total moves, average income percentile, and disadvantage index remain significant. Sex, race, marital status, homeownership, education, and the income×exposure interaction are not significant.

**Model Set 3 (Distance median/tail, Appendix Table 4):** Distance exposure (km) is significant in the base model and remains significant for mid-level exposure after adjusting for individual and household covariates. In fully adjusted models and models with the interaction term, the exposure effect is attenuated to non-significance. Age, total moves, average income percentile, and disadvantage index remain significant. Sex, race, marital status, homeownership, education, and the income×exposure interaction are not significant.

**Model Set 4 (Log-transformed pounds, Appendix Table 5:** Similar pattern to our main model, but the 4th quartile of exposure shows the highest (and statistically significant) OR for ADRD risk in models 1–3. In model 4, adding the interaction term attenuates the exposure effect to non-significance. Age, total moves, average income percentile, and disadvantage index remain significant. Sex, race, marital status, homeownership, and education are not significant.

The significant finding in the 4th quartile should be interpreted with caution. Log-transforming pounds before creating quartiles compresses high exposure values, meaning the 4th quartile encompasses an extremely wide range of original exposure levels. This broad categorization may mask important distinctions between moderately high and extremely high exposures and makes it difficult to determine whether the observed effect is driven by the full quartile or only the most extreme values within it.

**Model Set 5 (Log-transformed distance, Appendix Table 6):** No significant relationship between logged kilometer quartiles and ADRD risk. Age, total moves, average income percentile, and disadvantage index remain significant. Sex, race, marital status, homeownership, and education are not significant.

# E. References

1. Kind AJH, Buckingham WR. Making neighborhood disadvantage metrics accessible: the Neighborhood Atlas. N Engl J Med. 2018;378(26):2456-2458. doi:10.1056/NEJMp1802313
2. Stata Corp. PCA postestimation—Postestimation tools for pca and pcamat. In: Stata 19 Multivariate Statistics Reference Manual. StataCorp LLC; 2025. Accessed February 6, 2026. https://www.stata.com/manuals/mvpcapostestimation.pdf
