# Supplementary material for: Long‐Term Industrial Emissions Exposure and Cognitive Impairment in Older U.S. Adults: A Longitudinal Cohort Study
Source: Health Sci Rep. 2026 Sep 3;9(9):e73185. doi: 10.1002/hsr2.73185 (PMC13542189; doi:10.1002/hsr2.73185)
Supplement: Supplementary file 3 [file HSR2-9-e73185-s003.docx]

**Appendix Figure 1.** Scree plot of eigenvalues from principal component analysis (PCA) of eight tract-level disadvantage variables from the NCDB. The first component (eigenvalue = 3.33) explains the majority of shared variance, with a sharp drop to the second (1.68), supporting a one-factor solution. Data: NCDB, 1990–2020.
