# Supplementary material for: Long‐Term Industrial Emissions Exposure and Cognitive Impairment in Older U.S. Adults: A Longitudinal Cohort Study
Source: Health Sci Rep. 2026 Sep 3;9(9):e73185. doi: 10.1002/hsr2.73185 (PMC13542189; doi:10.1002/hsr2.73185)
Supplement: Supplementary file 5 [file HSR2-9-e73185-s005.docx]

**Eight Item Interview to Differentiate Aging and Dementia (AD8) Items in the PSID**

Next, we’d like to know about changes that [you / [target person]] may have had in the last several years
[Target person is not respondent: because of a thinking or memory problem].

In the last several years, has there been a change in:

1. Problems [you / he / she] may have with judgment, for example, problems making decisions, making bad financial decisions, or with thinking?

1. Yes, a change

5. No, no change

2. The amount of interest [you / he / she] may have in hobbies or activities?

1. Yes, a change

5. No, no change

3. [Your / His / Her] repeating the same things over and over, such as questions, stories, or statements?

1. Yes, a change

5. No, no change

4. The trouble [you / he / she] may have learning how to use a tool, appliance or gadget like a TV remote?

1. Yes, a change

5. No, no change

5. [Your / His / Her] forgetting the correct month or year?

1. Yes, a change

5. No, no change

6. The trouble [you / he / she] may have handling complicated money matters, like balancing a checkbook or paying bills?

1. Yes, a change

5. No, no change

7. The trouble [you / he / she] may have remembering appointments?

1. Yes, a change

5. No, no change

8. Daily problems [you / he / she] may have with thinking and/or memory?

1. Yes, a change

5. No, no change

Appendix Table 1. The eight-item AD8 dementia screening instrument. This table lists all eight questions from the AD8, a brief informant-based screening tool used to detect early signs of cognitive impairment. Each item reflects change in memory, orientation, judgment, or daily function. Responses are binary (yes/no), with endorsement of two or more items indicating possible cognitive decline. Copyright 2008 by Washington University in St. Louis, Missouri.
