# Supplementary material for: Long‐Term Industrial Emissions Exposure and Cognitive Impairment in Older U.S. Adults: A Longitudinal Cohort Study
Source: Health Sci Rep. 2026 Sep 3;9(9):e73185. doi: 10.1002/hsr2.73185 (PMC13542189; doi:10.1002/hsr2.73185)
Supplement: Supplementary file 6 [file HSR2-9-e73185-s006.docx]

|  | **Model 1** | **Model 2** | **Model 3** | **Model 4** |
| --- | --- | --- | --- | --- |
| **Variable** |  |  |  |  |
| **Logged Average Distance to Nearest Plastics/Rubber TRI Facility, km (1990-2017) *categorical, quartiles*** |  |  |  |  |
| *Ref = Q1* |  |  |  |  |
| Q2 | 0.807 | 0.876 | 0.912 | 1.081 |
| Q3 | 1.01 | 1.154 | 1.22 | 1.301 |
| Q4 | 0.948 | 0.901 | 0.956 | 1.135 |
|  |  |  |  |  |
| **Sex (2023)** |  |  |  |  |
| *Ref = Male* |  |  |  |  |
| Female |  | 0.899 | 0.903 | 0.927 |
|  |  |  |  |  |
| **Race (2023)** |  |  |  |  |
| *Ref = White* |  |  |  |  |
| Black |  | 1.048 | 0.925 | 0.986 |
| All other |  | 1.438 | 1.349 | 1.335 |
|  |  |  |  |  |
| **Age (2023)** |  |  |  |  |
| *Ref = 65-70 y.o.* |  |  |  |  |
| 71-80 y.o. |  | 2.021*** | 2.054*** | 2.072*** |
| 81-90 y.o. |  | 3.830*** | 3.933*** | 4.200*** |
| 91+ y.o. |  | 11.384*** | 12.258*** | 13.465*** |
|  |  |  |  |  |
| **Household Average Income Percentile (1990-2017)** |  | 0.981*** | 0.982*** |  |
|  |  |  |  |  |
| **Total Moves (1990-2017)** |  |  |  |  |
| *Ref = 0 moves* |  |  |  |  |
| 1-2 |  | 1.187 | 1.19 | 1.187 |
| 3-5 |  | 1.218 | 1.205 | 1.232 |
| 6-9 |  | 1.439* | 1.410* | 1.469* |
| 10+ |  | 2.204** | 2.201** | 2.543** |
|  |  |  |  |  |
| **Wtr Ever Married (2023)** |  |  |  |  |
| *Ref = Never married* |  |  |  |  |
| Current/previously married |  | 0.896 | 0.881 | 0.748 |
|  |  |  |  |  |
| **Home Ownership (1990-2017)** |  |  |  |  |
| *Ref = Never a homeowner* |  |  |  |  |
| Current or previous homeowner |  | 1.127 | 1.185 | 1.02 |
|  |  |  |  |  |
| **Max Education Level (1990-2017)** |  |  |  |  |
| *Ref = Less than 12 years* |  |  |  |  |
| 12 years or more, less than 16 years |  | 0.831 | 0.865 | 0.778 |
| 16 years or more |  | 0.837 | 0.871 | 0.695* |
|  |  |  |  |  |
| **Mean Disadvantage Index, Standardized (1990-2017)** |  |  | 1.179* | 1.231** |
|  |  |  |  |  |
| **Household Income Binary (recode of Household Average Income Percentile, 1990-2017)** |  |  |  |  |
| *Ref = Bottom 50%* |  |  |  |  |
| Top 50% |  |  |  | 0.714 |
|  |  |  |  |  |
| **Binary Income*Exposure to Emissions** |  |  |  |  |
| *Ref = Bottom 50% income*None* |  |  |  |  |
| Top 50% income*Q2 |  |  |  | 0.644 |
| Top 50% income*Q3 |  |  |  | 0.852 |
| Top 50% income*Q4 |  |  |  | 0.752 |
|  |  |  |  |  |
| _cons | 0.366*** | 0.534* | 0.462* | 0.323*** |
| n | 2627 | 2627 | 2627 | 2627 |
| aic | 2991.621 | 2755.3 | 2751.62 | 2770.894 |
| bic | 3015.115 | 2866.898 | 2869.092 | 2905.987 |
| ll | -1491.81 | -1358.65 | -1355.81 | -1362.447 |
| Appendix Table 6: Log-transformed km exposure divided into quartiles. | | | | |
