# Supplementary material for: Long‐Term Industrial Emissions Exposure and Cognitive Impairment in Older U.S. Adults: A Longitudinal Cohort Study
Source: Health Sci Rep. 2026 Sep 3;9(9):e73185. doi: 10.1002/hsr2.73185 (PMC13542189; doi:10.1002/hsr2.73185)
Supplement: Supplementary file 7 [file HSR2-9-e73185-s007.docx]

|  | **Model 1** | **Model 2** | **Model 3** | **Model 4** |
| --- | --- | --- | --- | --- |
| **Variable** |  |  |  |  |
| **Logged Average Exposure to Nearest Plastics/Rubber TRI Emissions, lbs (1990-2017) *categorical, quartiles*** |  |  |  |  |
| *Ref = Q1* |  |  |  |  |
| Q2 - omitted | - - - | - - - | - - - | - - - |
| Q3 | 1.185 | 1.153 | 1.148 | 1.111 |
| Q4 | 1.362** | 1.274* | 1.275* | 1.211 |
|  |  |  |  |  |
| **Sex (2023)** |  |  |  |  |
| *Ref = Male* |  |  |  |  |
| Female |  | 0.898 | 0.902 | 0.925 |
|  |  |  |  |  |
| **Race (2023)** |  |  |  |  |
| *Ref = White* |  |  |  |  |
| Black |  | 1.066 | 0.938 | 0.999 |
| All other |  | 1.438 | 1.344 | 1.336 |
|  |  |  |  |  |
| **Age (2023)** |  |  |  |  |
| *Ref = 65-70 y.o.* |  |  |  |  |
| 71-80 y.o. |  | 2.031*** | 2.065*** | 2.078*** |
| 81-90 y.o. |  | 3.870*** | 3.975*** | 4.223*** |
| 91+ y.o. |  | 11.563*** | 12.342*** | 13.242*** |
|  |  |  |  |  |
| **Household Average Income Percentile (1990-2017)** |  | 0.982*** | 0.983*** |  |
|  |  |  |  |  |
| **Total Moves (1990-2017)** |  |  |  |  |
| *Ref = 0 moves* |  |  |  |  |
| 1-2 |  | 1.186 | 1.191 | 1.188 |
| 3-5 |  | 1.185 | 1.173 | 1.201 |
| 6-9 |  | 1.371 | 1.349 | 1.411* |
| 10+ |  | 2.010* | 2.010* | 2.318** |
|  |  |  |  |  |
| **Wtr Ever Married (2023)** |  |  |  |  |
| *Ref = Never married* |  |  |  |  |
| Current/previously married |  | 0.886 | 0.874 | 0.752 |
|  |  |  |  |  |
| **Home Ownership (1990-2017)** |  |  |  |  |
| *Ref = Never a homeowner* |  |  |  |  |
| Current or previous homeowner |  | 1.094 | 1.156 | 1.01 |
|  |  |  |  |  |
| **Max Education Level (1990-2017)** |  |  |  |  |
| *Ref = Less than 12 years* |  |  |  |  |
| 12 years or more, less than 16 years |  | 0.823 | 0.854 | 0.766 |
| 16 years or more |  | 0.847 | 0.877 | 0.699* |
|  |  |  |  |  |
| **Mean Disadvantage Index, Standardized (1990-2017)** |  |  | 1.171* | 1.208** |
|  |  |  |  |  |
| **Household Income Binary (recode of Household Average Income Percentile, 1990-2017)** |  |  |  |  |
| *Ref = Bottom 50%* |  |  |  |  |
| Top 50% |  |  |  | 0.550*** |
|  |  |  |  |  |
| **Binary Income*Exposure to Emissions** |  |  |  |  |
| *Ref = Bottom 50% income*Q1* |  |  |  |  |
| Top 50% income*Q3 |  |  |  | 1.109 |
| Top 50% income*Q4 |  |  |  | 1.17 |
|  |  |  |  |  |
| _cons | 0.308*** | 0.485* | 0.437** | 0.345*** |
| n | 2627 | 2627 | 2627 | 2627 |
| aic | 2984.543 | 2753.81 | 2750.39 | 2769.402 |
| bic | 3002.164 | 2859.535 | 2861.989 | 2892.748 |
| ll | -1489.271 | -1358.905 | -1356.195 | -1363.701 |
| Appendix Table 5: Log-transformed lbs exposure divided into quartiles. | | | | |
