# Supplementary material for: Long‐Term Industrial Emissions Exposure and Cognitive Impairment in Older U.S. Adults: A Longitudinal Cohort Study
Source: Health Sci Rep. 2026 Sep 3;9(9):e73185. doi: 10.1002/hsr2.73185 (PMC13542189; doi:10.1002/hsr2.73185)
Supplement: Supplementary file 8 [file HSR2-9-e73185-s008.docx]

| **Variable** | **Model 1** | **Model 2** | **Model 3** | **Model 4** | **Model 5** |  |
| --- | --- | --- | --- | --- | --- | --- |
| **Average Distance to Nearest Plastics/Rubber TRI Facility, km (1990-2017) *categorical*** |  |  |  |  |  |  |
| *Ref = 1 Near, 0.53-30.54 km (min up to 75th percentile)* |  |  |  |  |  |  |
| 2 Moderate, 30.54-50.59 km (75th-90th percentile) | 0.956 | 0.879 | 0.887 | 0.968 | 0.88 |  |
| 3 Far, 50.59+ km (90th+ percentile) | 1.094 | 0.906 | 0.955 | 1.071 | 0.96 |  |
|  |  |  |  |  |  |  |
| **Sex (2023)** |  |  |  |  |  |  |
| *Ref = Male* |  |  |  |  |  |  |
| Female |  | 0.897 | 0.901 | 0.925 | 0.904 |  |
|  |  |  |  |  |  |  |
| **Race (2023)** |  |  |  |  |  |  |
| *Ref = White* |  |  |  |  |  |  |
| Black |  | 1.032 | 0.914 | 0.972 | 0.924 |  |
| All other |  | 1.405 | 1.313 | 1.298 | 1.33 |  |
|  |  |  |  |  |  |  |
| **Age (2023)** |  |  |  |  |  |  |
| *Ref = 65-70 y.o.* |  |  |  |  |  |  |
| 71-80 y.o. |  | 2.029*** | 2.063*** | 2.076*** | 2.073*** |  |
| 81-90 y.o. |  | 3.819*** | 3.926*** | 4.184*** | 3.984*** |  |
| 91+ y.o. |  | 11.163*** | 11.914*** | 12.892*** | 12.385*** |  |
|  |  |  |  |  |  |  |
| **Household Average Income Percentile (1990-2017)** |  | 0.981*** | 0.982*** |  | 0.983*** |  |
|  |  |  |  |  |  |  |
| **Total Moves (1990-2017)** |  |  |  |  |  |  |
| *Ref = 0 moves* |  |  |  |  |  |  |
| 1-2 |  | 1.19 | 1.196 | 1.196 | 1.194 |  |
| 3-5 |  | 1.22 | 1.212 | 1.246 | 1.172 |  |
| 6-9 |  | 1.440* | 1.419* | 1.484* | 1.354 |  |
| 10+ |  | 2.223** | 2.222** | 2.539** | 2.024* |  |
|  |  |  |  |  |  |  |
| **Wtr Ever Married (2023)** |  |  |  |  |  |  |
| *Ref = Never married* |  |  |  |  |  |  |
| Current/previously married |  | 0.89 | 0.878 | 0.75 | 0.874 |  |
|  |  |  |  |  |  |  |
| **Home Ownership (1990-2017)** |  |  |  |  |  |  |
| *Ref = Never a homeowner* |  |  |  |  |  |  |
| Current or previous homeowner |  | 1.14 | 1.196 | 1.032 | 1.168 |  |
|  |  |  |  |  |  |  |
| **Max Education Level (1990-2017)** |  |  |  |  |  |  |
| *Ref = Less than 12 years* |  |  |  |  |  |  |
| 12 years or more, less than 16 years |  | 0.826 | 0.854 | 0.766 | 0.843 |  |
| 16 years or more |  | 0.833 | 0.861 | 0.683* | 0.869 |  |
|  |  |  |  |  |  |  |
| **Mean Disadvantage Index, Standardized (1990-2017)** |  |  | 1.168* | 1.218** | 1.168* |  |
|  |  |  |  |  |  |  |
| **Household Income Binary (recode of Household Average Income Percentile, 1990-2017)** |  |  |  |  |  |  |
| *Ref = Bottom 50%* |  |  |  |  |  |  |
| Top 50% |  |  |  | 0.589*** |  |  |
|  |  |  |  |  |  |  |
| **Binary Income*Exposure to Emissions** |  |  |  |  |  |  |
| *Ref = Bottom 50% income*Low* |  |  |  |  |  |  |
| Top 50% income*Moderate |  |  |  | 0.909 |  |  |
| Top 50% income*High |  |  |  | 0.929 |  |  |
|  |  |  |  |  |  |  |
| **Average Exposure to Nearest Plastics/Rubber TRI Emissions, lbs (1990-2017) *categorical*** |  |  |  |  |  |  |
| *Ref = 0 None, 0 lbs* |  |  |  |  |  |  |
| 1 Low, >0-7627.52 lbs (up to 75th percentile) |  |  |  |  | 1.141 |  |
| 2 Moderate, 7627.52 lbs - 75797.66 lbs (75th-90th percentile) |  |  |  |  | 1.343* |  |
| 3 High, 75797.66+ lbs (90th+ percentile) |  |  |  |  | 1.202 |  |
|  |  |  |  |  |  |  |
| _cons | 0.343*** | 0.541* | 0.485* | 0.362** | 0.452* |  |
|  | **m1** | **m2** | **m3** | **m4** | **m5** |  |
| n | 2627 | 2627 | 2627 | 2627 | 2627 |  |
| aic | 2992.985 | 2757.355 | 2754.23 | 2774.362 | 2754.968 |  |
| bic | 3010.606 | 2863.08 | 2865.828 | 2897.708 | 2884.187 |  |
| ll | -1493.492 | -1360.678 | -1358.115 | -1366.181 | -1355.484 |  |
| Appendix Table 2. Distance (km) based replication of the five models presented in the paper. | | | | | |  |
|  |  |  |  |  |  |  |
