# Supplementary material for: Long‐Term Industrial Emissions Exposure and Cognitive Impairment in Older U.S. Adults: A Longitudinal Cohort Study
Source: Health Sci Rep. 2026 Sep 3;9(9):e73185. doi: 10.1002/hsr2.73185 (PMC13542189; doi:10.1002/hsr2.73185)
Supplement: Supplementary file 9 [file HSR2-9-e73185-s009.docx]

**Appendix Figure 2.** Distribution of the composite neighborhood disadvantage index derived from PCA of eight tract-level variables. Values are standardized (mean = 0, SD = 1). Data: NCDB, 1990–2020.
